# Supplementary material for: γ‐Secretase cleavage of the Alzheimer risk factor TREM2 is determined by its intrinsic structural dynamics
Source: EMBO J. 2020 Aug 24;39(20):e104247. doi: 10.15252/embj.2019104247 (PMC7560206; doi:10.15252/embj.2019104247)
Supplement: Supplementary file 1 — Appendix [file EMBJ-39-e104247-s001.pdf]

## Appendix to:

## γ-Secretase cleavage of the Alzheimer risk factor TREM2 is determined by its intrinsic structural dynamics

## Table of Contents

Appendix Figure S1 …………………………………………………………………………………… p.2

Appendix Figure S2 …………………………………………………………………………………… p.3

Appendix References ………………………………………………………………………………….. p.3

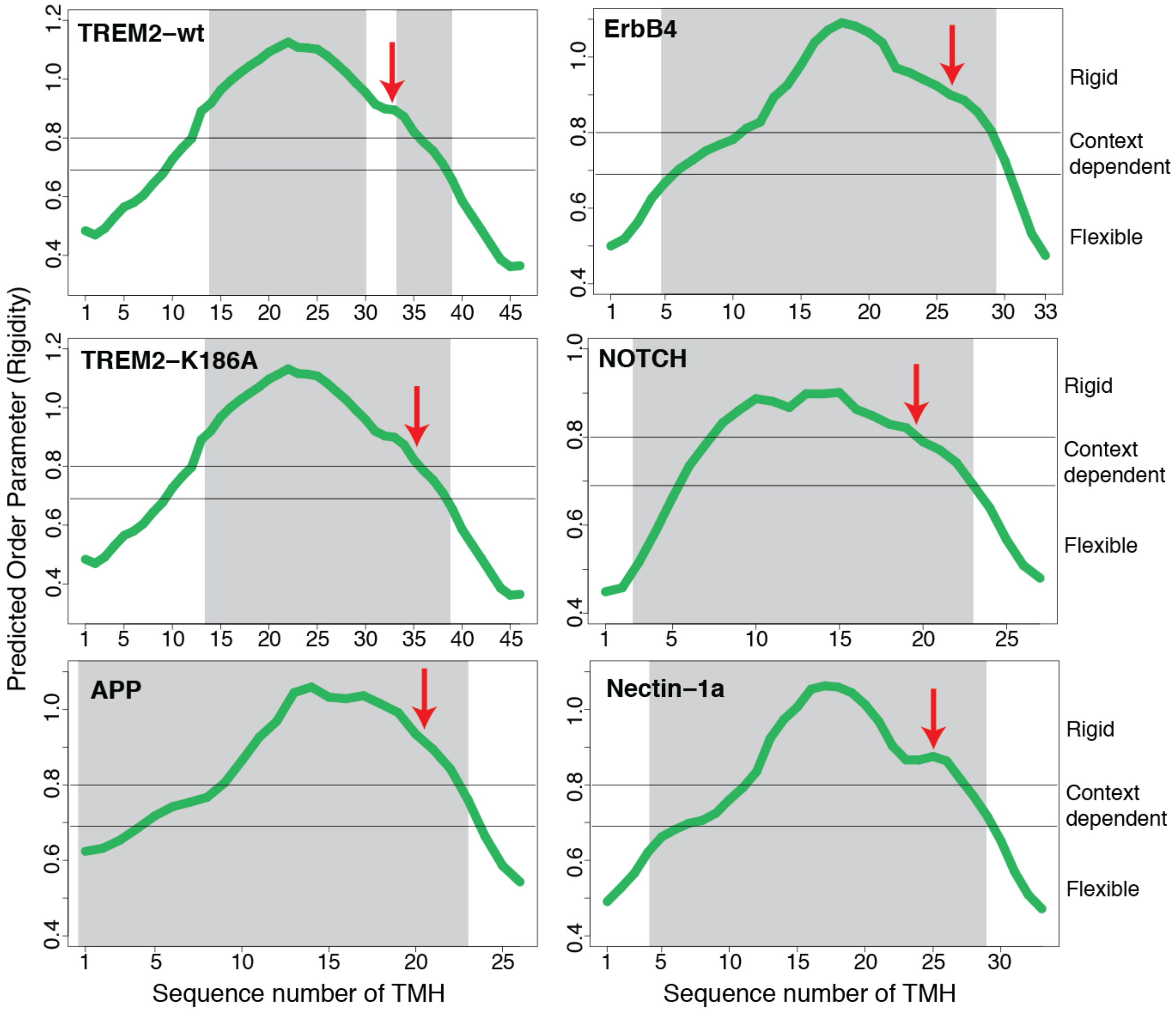


**Appendix Figure S1 - Estimation of TMH dynamics of various γ-secretase substrates.**

Sequence-based prediction of the order parameter (= rigidity) of selected proteins using the MemBrain online server (Yin *et al*, 2018). The position of the ε cleavage site of γ-secretase is taken from (Beel & Sanders, 2008) and is indicated by a red arrow. Grey shaded areas indicate predicted α-helical regions ine ach case. These patterns clearly show that initial γ-secretase cleavage takes place at approximately 3 residues distance from the C-terminal end of substrate helices where predicted order parameters are low. In the TREM2-TMH case, this distance is larger due to helix destabilization induced by the presence of K186 in the membrane. The regular 3-residue pattern can again be observed for the TREM2-TMH K186A variant.

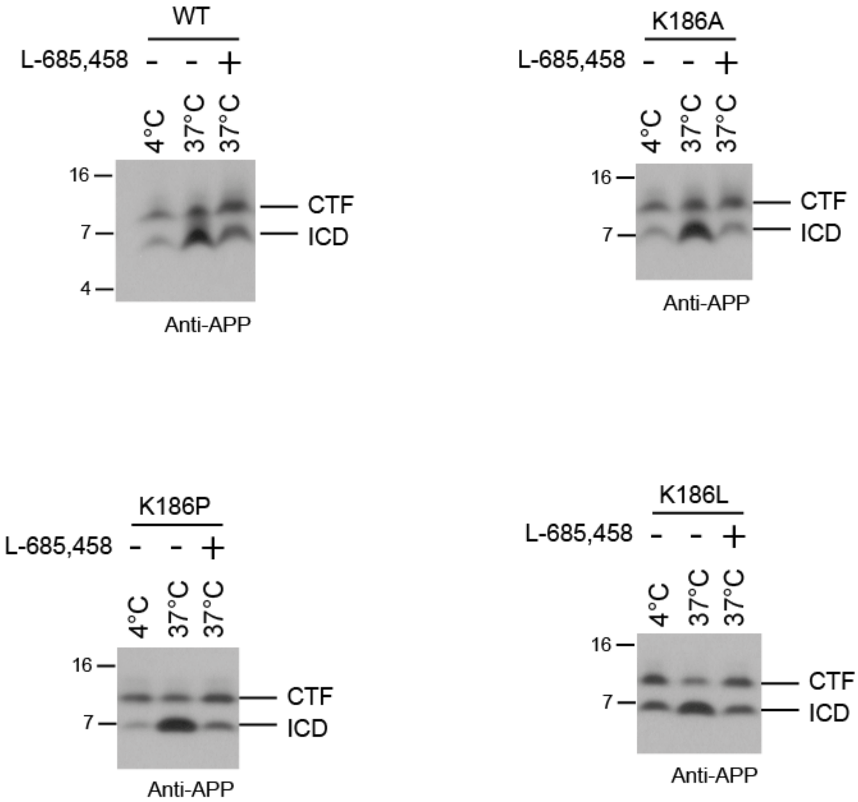


**Appendix Figure S2 - Immunoblot analysis of samples used for mass spectrometry.**

As a positive control for the mass spectrometric data shown in Fig. 5 immunoblot analyses of corresponding samples were conducted. Inhibition of APP ICD formation upon γ-secretase inhibition could be confirmed in all cases.

# Appendix References

Beel AJ, Sanders CR (2008) Substrate specificity of gamma-secreatse and other intramembrane proteases. *Cell Mol Life Sci* 65: 1311-1334

Yin X, Yang J, Xiao F, Yang Y, Shen HB (2018) MemBrain: An Easy-to-Use Online Webserver for Transmembrane Protein Structure Prediction. *Nanomicro Lett* 10: 2
