# Supplementary material for: γ‐Secretase cleavage of the Alzheimer risk factor TREM2 is determined by its intrinsic structural dynamics
Source: EMBO J. 2020 Aug 24;39(20):e104247. doi: 10.15252/embj.2019104247 (PMC7560206; doi:10.15252/embj.2019104247)
Supplement: Supplementary file 2 — Expanded View Figures PDF [file EMBJ-39-e104247-s002.pdf]

Expanded View Figures

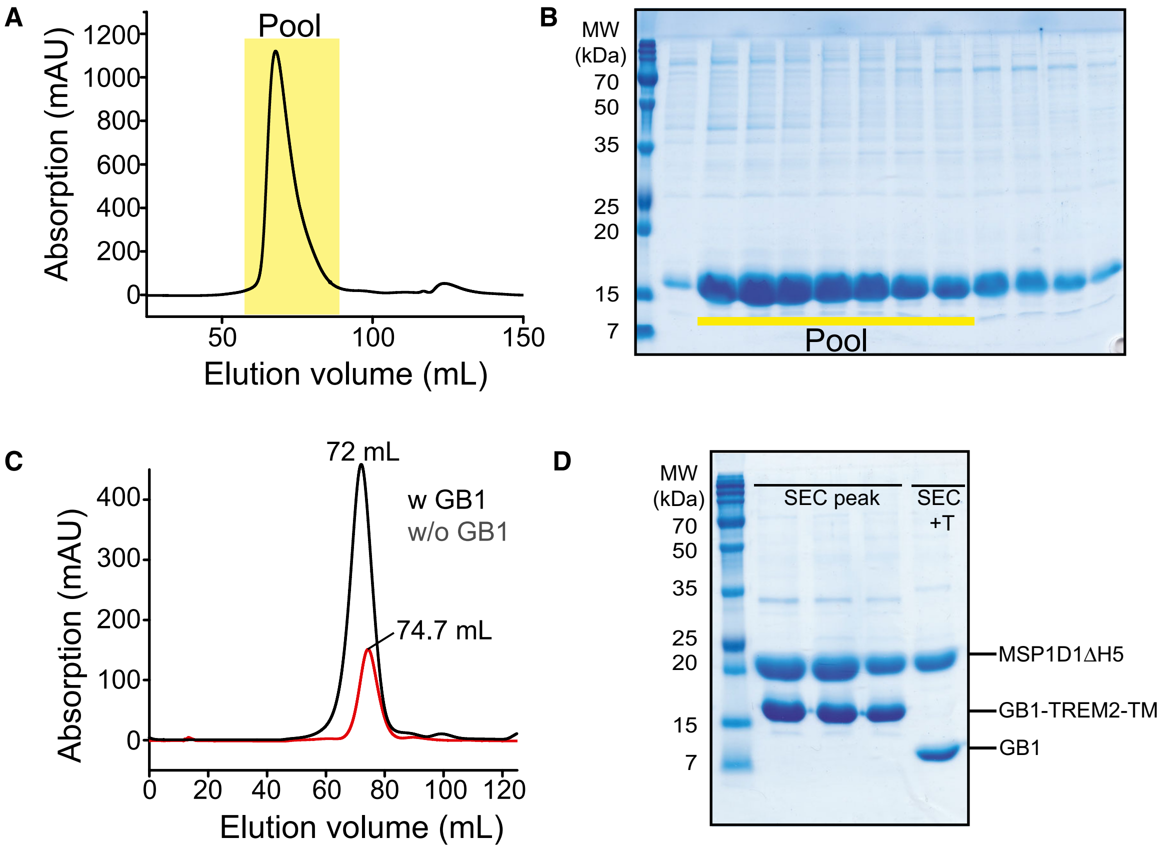

Figure EV1. Purification of TREM2-TMH in DPC micelles.

A Size exclusion chromatogram (SEC) of TREM2-TMH in DPC micelles using a Superdex200 column (124 ml bed volume). The indicated region of the chromatogram was pooled and used for NMR investigations.

B SDS–PAGE of SEC fractions containing TREM2-TMH.

C SEC chromatogram of MSP1D1ΔH5 nanodisc-incorporated TREM2-TMH fused to GB1 (black line) or after its removal by thrombin protease cleavage, followed by Ni-NTA chromatography (red line). The apparent increase in elution volume for the cleaved protein without GB1 indicates a reduction in size.

D SDS–PAGE of TREM2-TMH in nanodiscs before and after addition of thrombin protease for GB1 removal. T: thrombin. Due to its small size of ~5 kDa, TREM2-TMH is not visible on the gel. Co-elution of TREM2-TMH and the membrane scaffold protein (MSP) in the same SEC fractions indicates successful nanodisc incorporation.

Figure EV2. NMR structural investigations on TREM2-TMH in phospholipid nanodiscs. ▶

A 2D-[^{1}H,^{15}N]-TROSY spectrum at 37°C of *U*-^{2}H,^{13}C,^{15}N-labeled TREM2-TMH in nanodiscs composed of MSP1D1ΔH5 and DMPC/DMPG (3:1) lipids. Assigned backbone amide resonances are labeled, as obtained with 3D-HNCA and 3D-NOESY spectra.

B Cα secondary chemical shifts of TREM2-TMH in nanodiscs indicate α-helical secondary structure (positive values) between residues 173 and 198. Cα chemical shift information could not be obtained for this sample due to sensitivity issues. No data could be obtained for proline stretches and regions where no signal could be observed in the TROSY spectrum.

C 3D-^{15}N-edited-[^{1}H,^{1}H]-NOESY spectra show that the transmembrane helical conformation is interrupted by an unstructured stretch between residues 189–192, as indicated by a lack of sequential contacts between amide protons (red broken lines).

D Overlay of 2D-[^{1}H,^{15}N]-TROSY spectra of TREM2-TMH in DPC micelles (black) and DMPC/DMPG (3:1) nanodiscs. The chemical shift perturbations (CSPs) of TREM2-TMH in DPC versus nanodiscs are plotted below the spectrum in red. Gray bars are CSP values in DPC micelles upon the addition of DAP12 for comparison.

E Overlay of 2D-[^{1}H,^{15}N]-TROSY spectra of TREM2-TMH in POPC/POPG (3:1) (black) and DMPC/DMPG (3:1) (red) nanodiscs. The CSPs of TREM2-TMH in DMPC/PG versus POPC/PG nanodiscs are plotted below the spectrum in red. Gray bars are CSP values in DPC micelles upon the addition of DAP12 for comparison.

 © 2020 The Authors

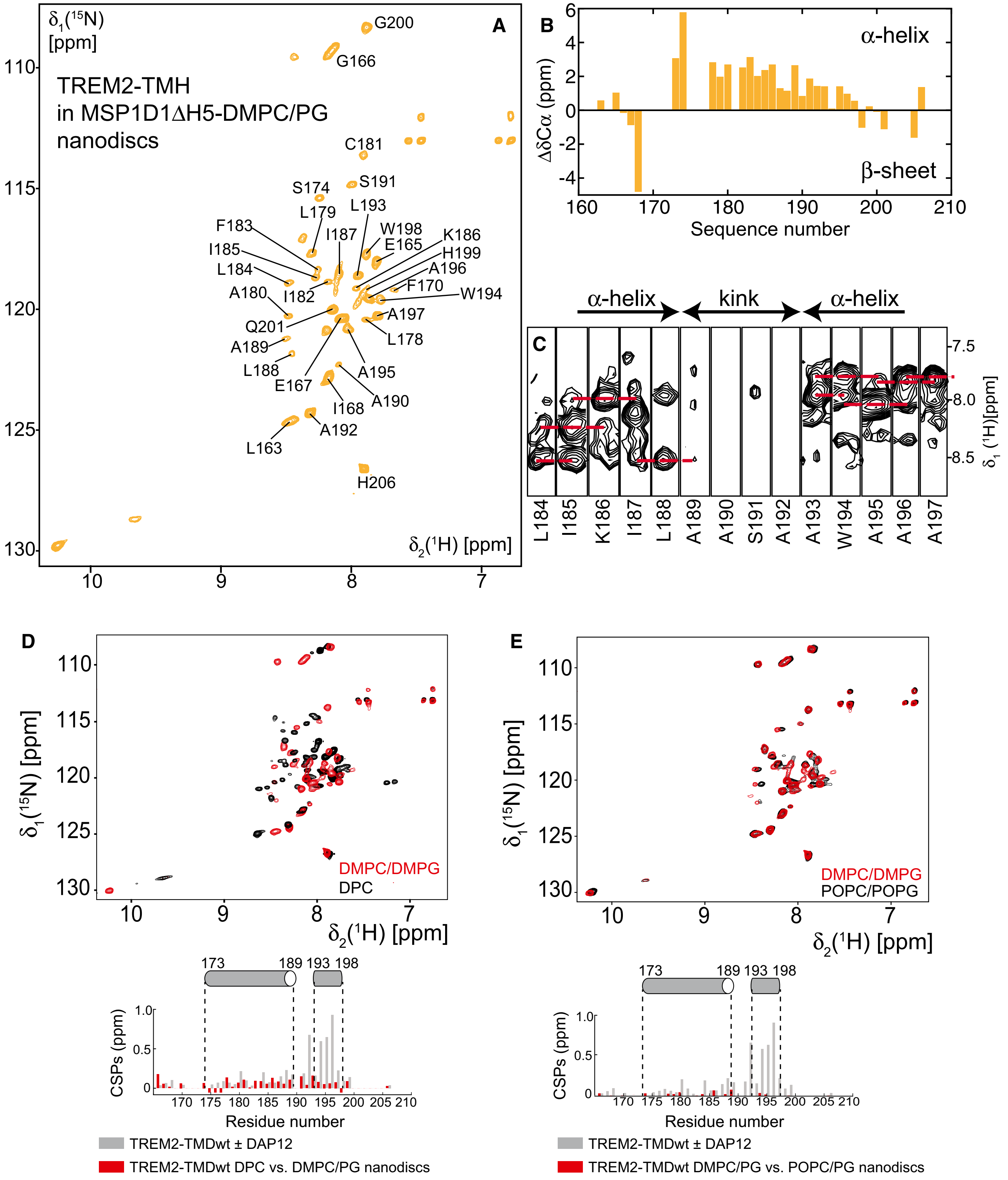
A
TREM2-TMH
in MSP1D1ΔH5-DMPC/PG
nanodiscs
δ1(15N) [ppm]
δ2(1H) [ppm]
G200
G166
C181
S174
S191
L179
L193
F183
I187
W198
K186
E165
I185
H199
L184
A196
F170
A180
I182
W194
A197
Q201
L178
A189
L188
A195
E167
A190
I168
A192
L163
H206
B
α-helix
β-sheet
ΔδCα (ppm)
Sequence number
C
α-helix
kink
α-helix
L184 I185 K186 I187 L188 A189 A190 S191 A192 A193 W194 A195 A196 A197
δ1 (1H)[ppm]
D
δ1(15N) [ppm]
δ2(1H) [ppm]
DMPC/DMPG
DPC
173 189 193 198
CSPs (ppm)
Residue number
TREM2-TMDwt ± DAP12
TREM2-TMDwt DPC vs. DMPC/PG nanodiscs
E
δ1(15N) [ppm]
δ2(1H) [ppm]
DMPC/DMPG
POPC/POPG
173 189 193 198
CSPs (ppm)
Residue number
TREM2-TMDwt ± DAP12
TREM2-TMDwt DMPC/PG vs. POPC/PG nanodiscs

Figure EV2.

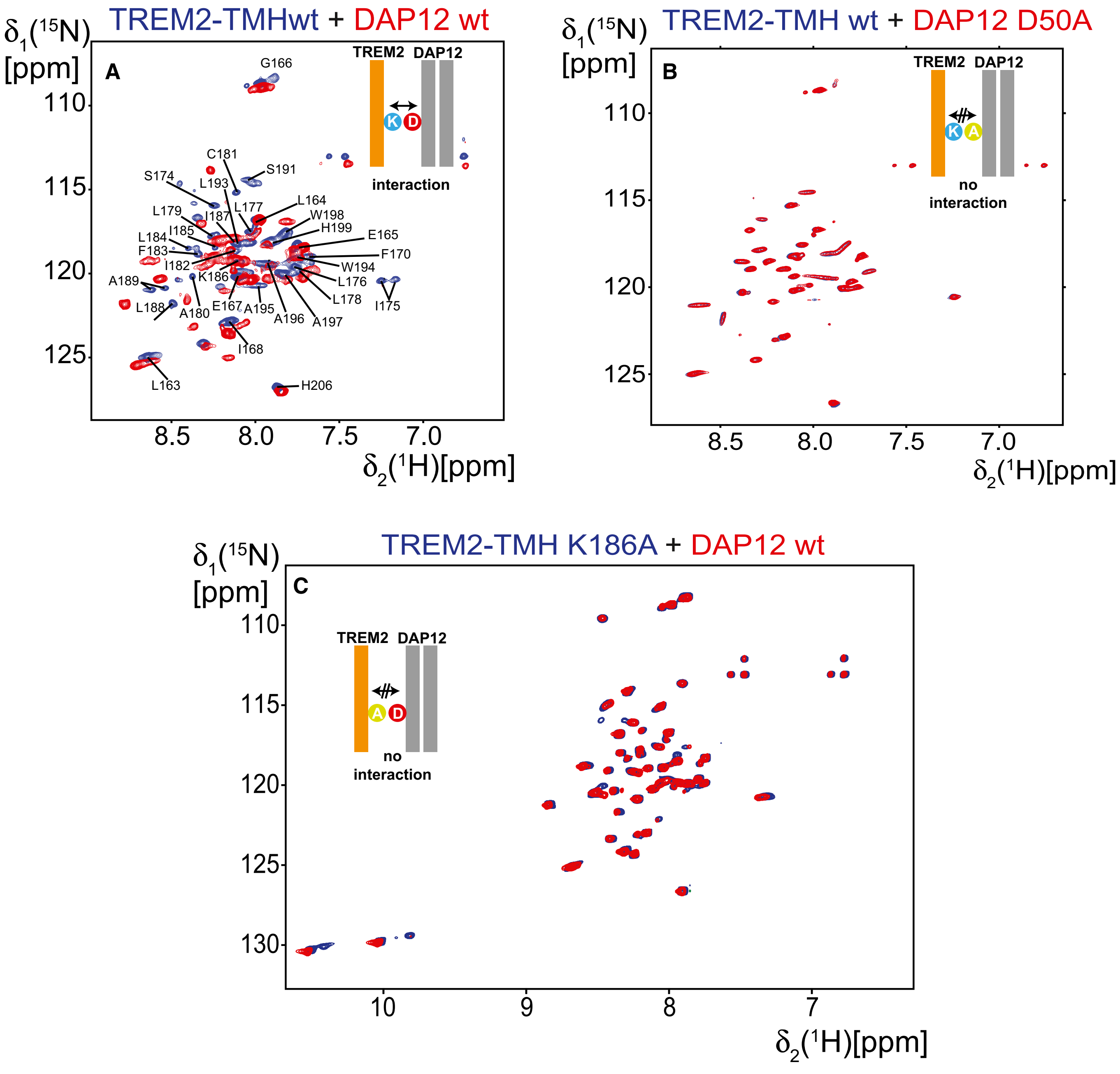

Figure EV3. Interaction between TREM2-TM and DAP12 probed by 2D-TROSY spectra.

A TREM2 interacts with DAP12 wild-type as can be seen by pronounced CSPs.

B In contrast, no CSPs are observed if the DAP12 D50A variant is added to *U*-^{2}H,^{15}N-labeled TREM2-TMH (right). Blue spectrum: TREM2-TMH, red spectrum: TREM2-TMH in complex with either DAP12-wt or the D50A variant.

C An identical picture can be observed if the charge is knocked out in TREM2-TMH by the K186A mutation with no spectral changes in isotope-labeled TREM2-TMH K186A upon addition of DAP12 wt.

© 2020 The Authors

Figure EV4. Membrane location of TREM2-TMH wt and K186 in detergent micelles and lipid nanodiscs.

A The Hε protons of the two tryptophan (W) residues in TREM2-TMH wt show distinct NOE contact patterns with the individual regions of the DPC molecule. For W194, strong NOE cross-peaks to the DPC methylene moieties can be observed, indicative of a membrane-inserted or -proximal location of this side chain. Contacts to the cholate group of DPC and the solvent are visible, too, corroborating a peripheral location. For W198, no signal to any part of DPC can be observed, indicative of a dynamic state further outside the micelle.

B For the TREM2-TMH K186A variant, strong contacts for both Trp residues to the inside of the micelle can be observed. Since W198 is located at the end of the TMH, its higher solvent accessibility gives rise to a stronger NOE signal to H_2O at 4.7 ppm.

C, D Strips of a 3D-^{15}N-edited-[^{1}H,^{1}H]-NOESY experiment of ^{2}H,^{15}N-labeled TREM2-TMH TREM2-TMH in DMPC/DMPG lipid nanodiscs. NOE cross-peaks of TREM2 backbone amides to specific regions of the lipid are indicated by broken lines. The corresponding structural features of the DMPC lipid molecule are shown in panel (D). The first α-helix (aa 173–189) of TREM2-TMH is showing contacts to methylene groups and the terminal methyl groups of the fatty acids, indicating an integral membrane location. The C-terminal helix (aa 193–198) is in a more membrane attached location, where NOE contacts are mostly restricted to methylene groups of the fatty acid or the lipid headgroup region.

E Model of TREM2-TMH in a lipid bilayer environment.

© 2020 The Authors

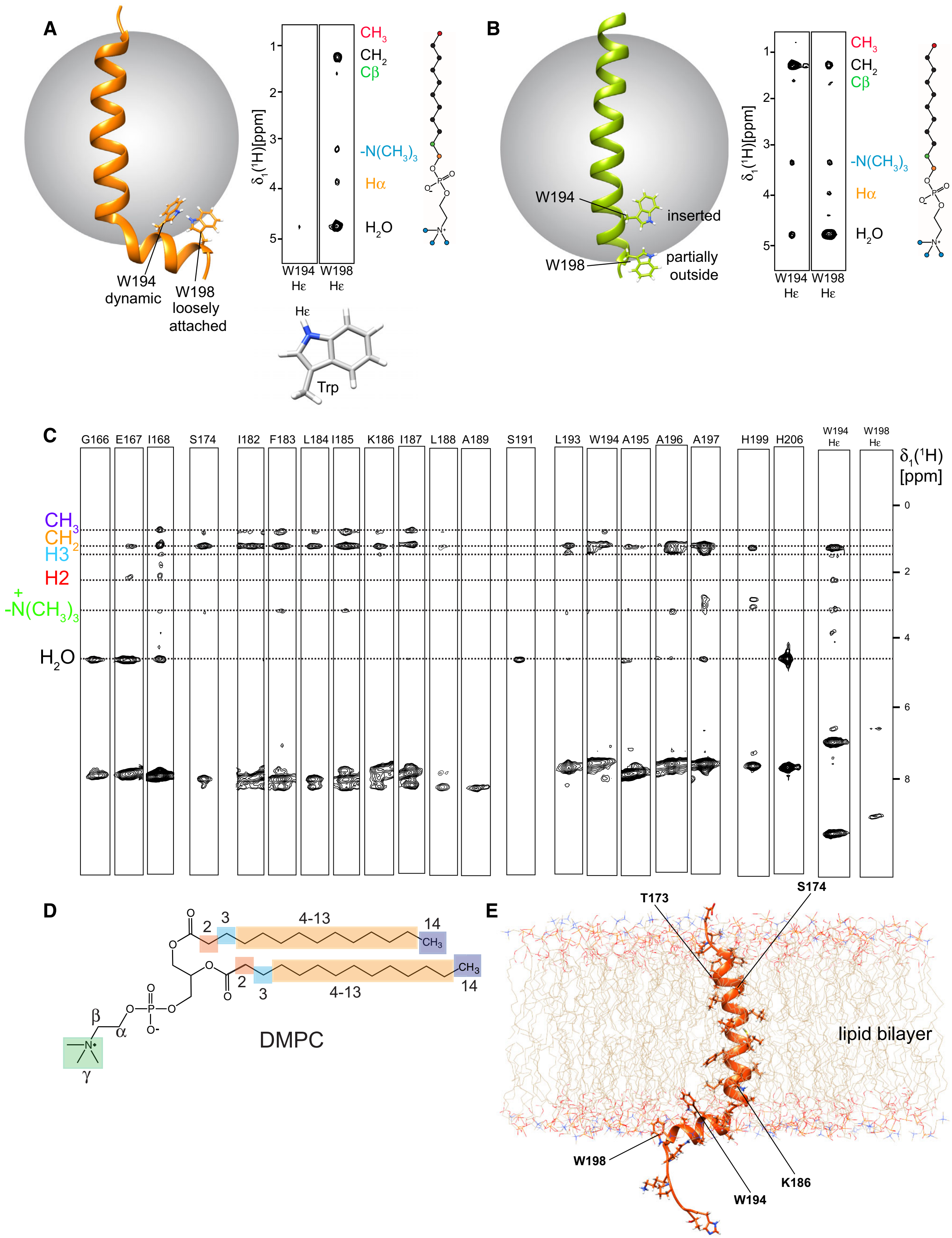

Figure EV4.

 © 2020 The Authors

Figure EV5. Deuterium exchange experiments of TREM2-TMH wt and K186A in DPC and lipid nanodiscs.

TREM2-TMH was solvent exchanged from H_2O based to D_2O-based buffer and 2D-[^1H,^{15}N]-TROSY experiments were recorded in each state at 310 K.

A With TREM2-TMH wt in DPC micelles, only the very central parts of the TMH is still visible in D_2O, suggesting that all other amino acid backbone amides are solvent accessible and/or are not participating in a stable hydrogen bond.
B Same as in (A) but using TREM2-TMH in lipid nanodiscs. A similar pattern of residual signal intensities can be observed as in DPC.
C TREM2-TMH K186A in DPC micelles. Here, more signals are still visible or unaffected in D_2O. Signals corresponding to almost the entire TMH are visible in that case.

© 2020 The Authors

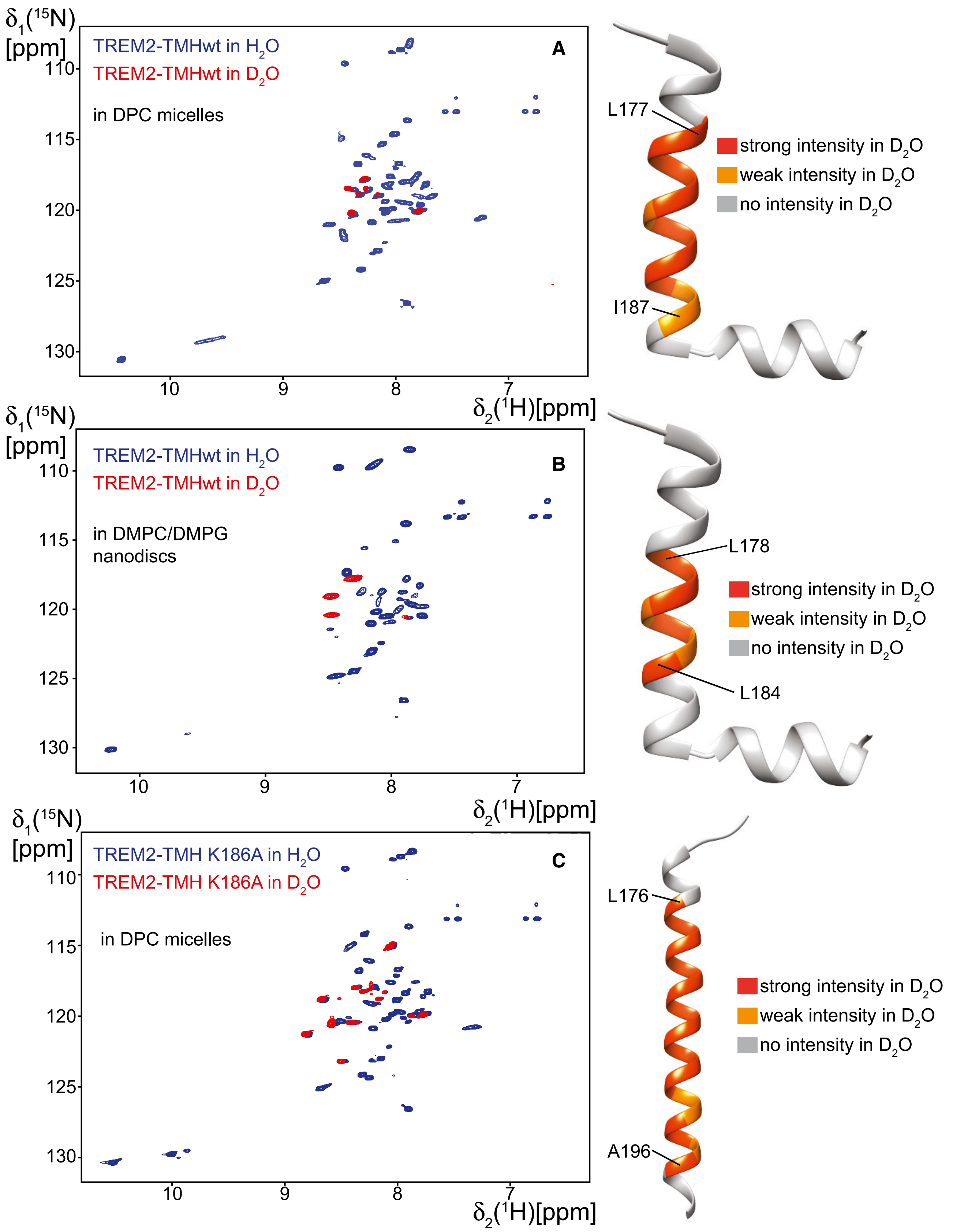

Figure EV5.

 © 2020 The Authors

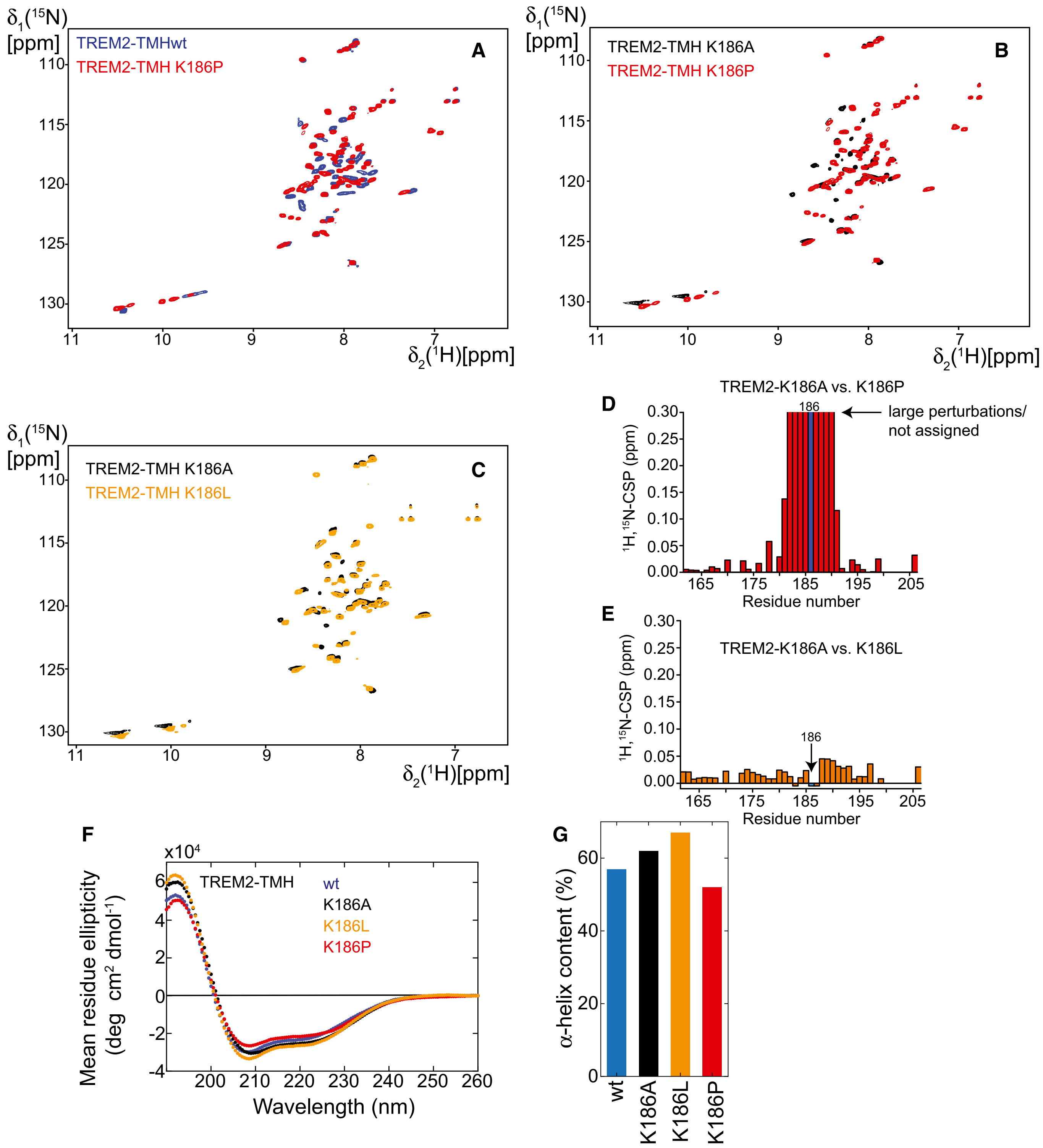

A
δ1(15N) [ppm]
TREM2-TMHwt
TREM2-TMH K186P
δ2(1H)[ppm]
B
TREM2-TMH K186A
TREM2-TMH K186P
C
TREM2-TMH K186A
TREM2-TMH K186L
D
TREM2-K186A vs. K186P
186
large perturbations/ not assigned
1H,15N-CSP (ppm)
Residue number
E
TREM2-K186A vs. K186L
F
x10⁴
TREM2-TMH
wt
K186A
K186L
K186P
Mean residue ellipticity (deg cm2 dmol-1)
Wavelength (nm)
G
α-helix content (%)
wt
K186A
K186L
K186P

Figure EV6.

Figure EV6. Investigation of structural changes within TREM2-TMH upon mutation of residue K186.

A, B Spectral overlay of 2D-[^{1}H,^{15}N]-TROSY experiments of (A) TREM2-TMH wt (blue) and the K186P (red) variant or (B) of TREM2-TMH K186A (black) and K186P (red) variants. The presence of a sterically demanding proline residue most likely induces a kink in the α-helical conformation leading to marked changes in the NMR spectra.

C Overlay of 2D-[^{1}H,^{15}N]-TROSY spectra of TREM2-TMH K186A (black) and K186L (yellow) shows no pronounced differences, indicating that a hydrophobic amino acid at position 186 leads to a stable secondary structure in the TMH.

D CSPs between the spectra shown in panel (B) are clustered around the site or the proline residue at position 186.

E CSPs between spectra shown in panel (C) are very small, suggesting a similar structure of both protein variants.

F CD spectra of the variants investigated by NMR, as indicated.

G The α-helical secondary structure content estimated by the MRW ellipticity at 222 nm in panel (F) (see Materials and Methods section) shows that TREM2-TMH wt and the K186P variant contain less secondary structure than the K186A or K186L variants.

 © 2020 The Authors
